# Clinical and Molecular Analysis of ST11-K47 Carbapenem-Resistant Hypervirulent *Klebsiella pneumoniae*: A Strain Causing Liver Abscess

**DOI:** 10.3390/pathogens11060657

**Published:** 2022-06-07

**Authors:** Zhen Cai, Tianye Jia, Mingfang Pu, Shuyong Zhang, Jingxia Zhang, Ronghua Geng, Suming Chen, Yahao Li, Huahao Fan, Yigang Tong, Fen Qu

**Affiliations:** 1 Aviation General Hospital, Beijing 100012, China; caizhencc@126.com (Z.C.); wabwabcde11@163.com (S.Z.); bjzjx361@163.com (J.Z.); grh361@163.com (R.G.); 2 The Clinical Laboratory, Fifth Medical Center of PLA General Hospital, Beijing 100039, China; jiatianye84@163.com (T.J.); chensuming987@163.com (S.C.); 3 College of Life Science and Technology, Beijing University of Chemical Technology, Beijing 100029, China; 2020210710@mail.buct.edu.cn; 4 Beijing Advanced Innovation Center for Soft Matter Science and Engineering (BAIC-SM), Beijing University of Chemical Technology, Beijing 100029, China; liyahao0036@163.com

**Keywords:** *Klebsiella pneumoniae*, liver abscess, hypervirulent, virulence genes, carbapenem resistance, ST11-K47

## Abstract

*Klebsiella pneumoniae* has been the predominant pathogen of liver abscess, but ST11-K47 carbapenem-resistant hypervirulent *Klebsiella pneumoniae* (CR-hvKP) has rarely been studied as the causative organism. We identified an ST11-K47 CR-hvKP (HvKp-su1) from the drainage fluid of a liver abscess in a Chinese man who was diagnosed with liver abscess combined with diabetes, pneumonia, pleural infection, abdominal abscess, and splenic abscess. HvKp-su1 was non-hypermucoviscous and lacked the *magA* and *rmpA* genes and pLVPK plasmid but exhibited high virulence, with a high mortality rate (90%) to wax moth larvae (*G. mellonella*), similar to the hypervirulent *Klebsiella pneumoniae* ATCC43816 (91.67%). Whole-genome sequencing and bioinformatics analysis indicated that HvKp-su1 possesses a plasmid similar to a type of pLVPK-like plasmid (JX-CR-hvKP-2-P2), which is an uncommon plasmid in CR-hvKP. HvKp-su1 carried multiple resistance genes, including *bla*_KPC-2_. *bla*_TEM-1_, *bla*_SHV-55_, and *bla*_CTX-M-65_; hypervirulence genes such as aerobactin (*iutA*), salmochelin (*iroEN*), and yersiniabactin (*ybtAEPQSTUX*); and the type 3 fimbriae-encoding system (*mrkACDF*). Moreover, v_5377 and v_5429 (*cofT,* CFA/III (CS8)) located on plasmid 1 were simultaneously predicted to be virulence genes. After the long-term combination use of antibiotics, the patient successfully recovered. In summary, our study clarified the clinical and molecular characteristics of a rare ST11-K47 CR-hvKP (HvKp-su1), raising great concerns about the emergence of ST11-K47 CR-hvKP with multidrug resistance and hypervirulence, and providing insights into the control and treatment of liver abscess caused by ST11-K47 CR-hvKP.

## 1. Introduction

Hypervirulent *Klebsiella pneumoniae* (hvKP) usually presents a positive “string test” (using an inoculation loop to generate a viscous string <5 mm from a bacterial colony), which is one of the features distinguishing it from classical *Klebsiella pneumoniae* (cKP). It is the main causative pathogen of liver abscess in East Asian countries and may cause other metastatic infections at the same time, including pneumonia, endophthalmitis, brain abscess, and other diseases [1,2,3]. Mucoviscosity-associated gene A (*magA*), hypermucoviscous phenotype regulator genes (*rmpA*/*rmpA2*), siderophore system aerobactin genes (*iut*, *iuc*), salmochelin genes (*iro*), and yersiniabactin genes (*ybt*, *irp*) have been identified as its critical virulence determinants [4,5,6,7]. Most hvKPs possess a large virulence plasmid (pLVPK), which carries virulence genes, such as aerobactin genes and *rmpA*, participating in their hypervirulence phenotypes [8,9].

ST23 are the dominant sequence type of hvKP strains, and K1 or K2 types are their most frequent capsule types [6]. To date, compared with the high prevalence of antibiotic resistance in cKP isolates, the majority of hvKP strains are antibiotic-sensitive [10,11,12,13]. However, by acquiring plasmids containing carbapenem resistance genes of hvKP or the acquisition of virulence plasmids by cKP, CR-hvKP has become a new threat to the world [10,14].

Although reports of CR-hvKP strains are increasing in China, the clinical and molecular characteristics of ST11-K47 CR-hvKP strains are not yet well understood. In this study, we conducted animal experiments and comparative genomic analysis to understand the virulence and antimicrobial resistance mechanisms of an ST11-K47 CR-hvKP strain isolated from a patient with a serious liver abscess.

## 2. Results

### 2.1. Patient Information and Clinical Characteristics

**Figure 1:**
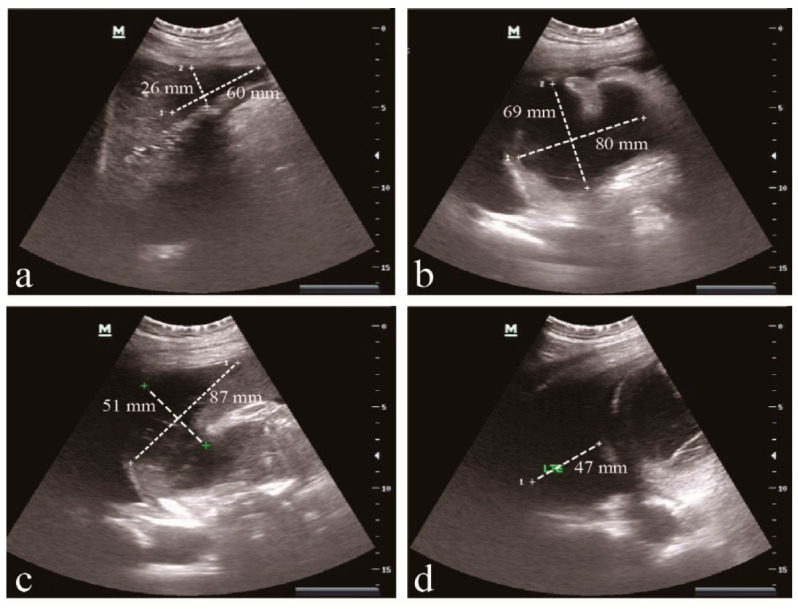
Abdominal ultrasonography. (**a**) The hepatic abscess (60 × 26 mm) was located in the liver’s left lobe. (**b**) Spleen fossa abscess (80 × 69 mm). (**c**) Anechoic area near the spleen (87 × 51 mm). (**d**) Pleural effusion on the left (47 mm).

**Figure 2:**
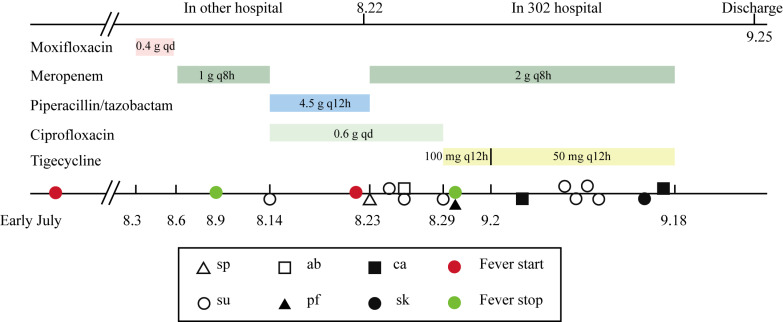
Timeline of antibiotic usage and *K. pneumoniae* isolation. Rectangles of various colors represent the time of antibiotic usage. Triangles, squares, and circles represent the time of *K. pneumoniae* isolation from different specimens. The red circle represents the time of fever initiation, and the green circle represents the time of fever cessation. sp: sputum, ab: abdominal fluid, su: shunt fluid, pf: pleural fluid, ca: catheter, sk: skin.

HvKp-su1 was cultured from the shunt fluid of a liver abscess in a 67-year-old Chinese man. This patient presented with a high fever and chills that lasted intermittently for approximately one month. He was diagnosed with a primary hepatic abscess combined with ascites, primary peritonitis, pleural effusion, type II diabetes, splenic abscess, and anemia (Figure 1). During approximately one month of hospitalization in the Fifth Medical Center of PLA General Hospital (302 Hospital), carbapenem-resistant *Klebsiella pneumoniae* (CR-KP) were isolated from shunt fluid, abdominal fluid, catheters, pleural fluid, and sputum 13 times, and 6 times from shunt fluid of liver abscess, which demonstrated that HvKp-su1 was the causative agent of the liver abscess (Figure 2). Laboratory results performed on the first day of hospitalization at 302 Hospital are listed in Table 1.

### 2.2. Treatment and Outcomes

Before admission to 302 Hospital, this patient received a combination of moxifloxacin and meropenem plus liver abscess incision drainage. However, he had a poor outcome with a high fever again after a month of treatment. At 302 Hospital, drainage from the liver pus cavities was continued, and percutaneous drainage from the spleen fossa was also performed. The anti-infection therapy scheme was changed to meropenem combined with ciprofloxacin. Adjuvant therapy included the use of insulin, blood plasma, and a nutrient solution. One week later, the therapy program was adjusted to meropenem combined with tigecycline. After one month of hospital stay, all drainage tubes were removed, as a B-ultrasound showed that the liver abscess had disappeared and the splenic abscess was significantly reduced. The patient, who could self-care, was discharged and remained completely normal with a follow-up at 3 months.

In summary, this patient received a hospital stay of 56 days (22 days in another hospital and 34 days in 302 Hospital). He had an antibiotic duration of approximately 51 days (moxifloxacin, meropenem, piperacillin/tazobactam, ciprofloxacin, and tigecycline). Until his temperature returned to completely normal, this patient had an intermittent fever time of approximately two months (Figure 2).

### 2.3. Microbiological Characteristics of HvKp-su1

HvKp-su1 was identified as *K. pneumoniae* by Vitek II (bioMérieux, Marcy-l’Étoile, France), and antimicrobial susceptibility results indicated that it was resistant to amikacin (MIC, ≥64 μg/mL), cefepime (MIC, ≥32 μg/mL), ceftazidime (≥64 μg/mL), aztreonam (MIC, ≥64 μg/mL), ciprofloxacin (MIC, ≥4 μg/mL), levofloxacin (MIC, ≥8 μg/mL), imipenem (MIC, ≥16 μg/mL), meropenem (MIC, ≥16 μg/mL), and cefoperazone/sulbactam (MIC, ≥64 μg/mL), and susceptible to tigecycline (MIC, 2 μg/mL) and polymyxin B (MIC, ≤0.5 μg/mL). This result demonstrated that HvKp-su1 is a carbapenem-resistant *K. pneumoniae.*

To evaluate the hypermucoviscosity phenotype of strain HvKp-su1, we stretched bacterial colonies on an agar plate with an inoculation loop and found that the viscous string was <5 mm.

### 2.4. Virulence Test of HvKp-su1

**Figure 3:**
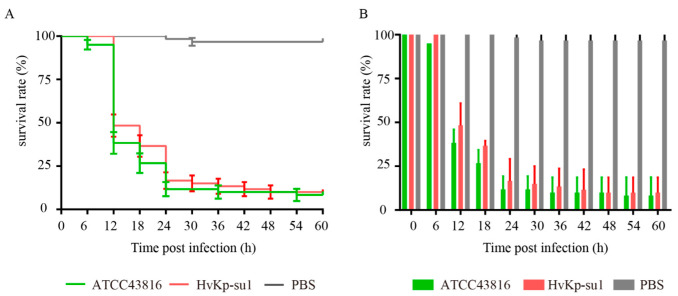
Survival rate of *G. mellonella* infected with *K. pneumoniae* strains. Larvae were injected with 4 μL PBS or with 2 × 10^6^ CFU/mL ATCC43816 (positive control) and HvKp-su1, and survival was monitored over 60 h post-infection. Survival rate was represented with survival curve (**A**) and column (**B**). This experiment was repeated in biological triplicate.

Since the “string test” performed less well as a marker for distinguishing hvKP and cKP, an in vivo virulence test of *G. mellonella* infection was also necessary. To evaluate the virulence of strain HvKp-su1, we infected *G. mellonella* larvae and observed their survival rates. Hypermucous K2 *K. pneumoniae* strain ATCC 43816 was used as the positive control for hvKP, and PBS was used as the negative control. Four microliters of 2 × 10^6^ CFU/mL strains or PBS were injected into 20 larvae, and survival was recorded every 6 h for 60 h. After 60 h of infection, the survival of *G. mellonella* larvae was 8.33% with strain ATCC43816, 10.00% with strain HvKp-su1, and 96.67% with PBS. This indicated that HvKp-su1 was hypervirulent (Figure 3).

### 2.5. MLST Genotyping of HvKp-su1 and Other K. pneumoniae

As *K. pneumoniae* were also separated from shunt fluid of the abdomen, spleen fossa, abdominal fluid, catheter, and pleural fluid, we supposed that those *K. pneumoniae* may have high homology. To better understand their genetic basis, HvKp-su1and 5 *K. pneumoniae* strains (HvKp-su2, HvKp-su3, HvKp-ab, HvKp-ca, HvKp-pf) were analyzed. MLST analysis revealed that the six *K. pneumoniae* isolates from different sites all belonged to ST11 (gapA-infB-mdh-pgi-phoE-rpoB-tonB allele number 3-3-1-1-1-1-4) and capsular type K47. Moreover, antimicrobial susceptibility results demonstrated that those five *K. pneumoniae* strains were all resistant to imipenem and meropenem.

### 2.6. Molecular Analysis of HvKp-su1

**Figure 4:**
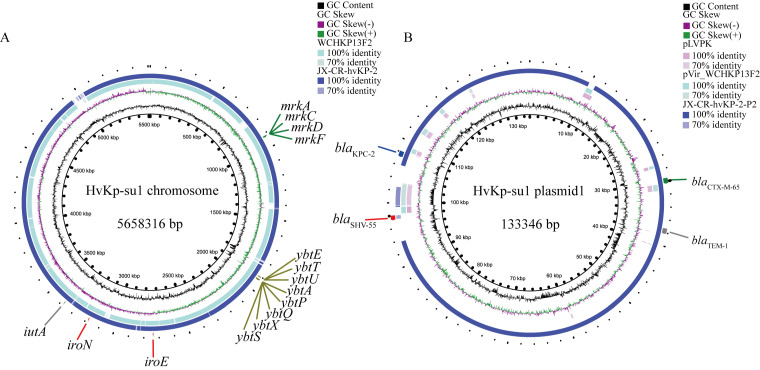
Gene map of chromosome HvKp-su1 and plasmid1 compared with genomes harbored by hvKP strains. (**A**) Alignment of WCHKP13F and JX-CR-hvKP-2 with HvKp-su1 chromosomes. (**B**) Alignment of pLVPK, pVir_WCHKP13F2, JX-CR-hvKP-2-P2 with HvKp-su1 plasmid1. JX-CR-hvKP-2 and JX-CR-hvKP-2-P2 are from ST11 CR-hvKP. WCHKP13F2 and pVir_WCHKP13F2 are from ST36 CR-hvKP. pLVPK is from ST23 CR-hvKP. The circular map was generated using the BLAST Ring Image Generator (BRIG). Accession numbers for the chromosome are CP028391 (WCHKP13F2) and CP064246 (JX-CR-hvKP-2). Accession numbers for the plasmids are MF943217 (pVir_WCHKP13F2), AY378100 (pLVPK), CP064248.1 (JX-CR-hvKP-2-P2).

We applied whole-genome sequencing to strain HvKp-su1 to reveal its genetic background. The genome size of strain HvKp-su1 was 5,658,316 bp, including one chromosome and two plasmids. Blastn online found that strain HvKp-su1 was quite similar to a CR-hvKP clinical isolate (*K. pneumoniae* JX-CR-hvKP-2). Plasmid 1 of HvKp-su1 has 99.97% identity with *K. pneumoniae* JX-CR-hvKP-2-P2 (E value: 0.0, Cover: 91%). The chromosome sequence of strain HvKp-su1 has 99.99% identity with JX-CR-hvKP-2 (E value: 0.0, Cover: 97%). Moreover, plasmid 1 of HvKp-su1 has poor homology with known virulent plasmids (pLVKP (GenBank AY378100) and pVir_WCHKP13F2 (GenBank MF943217)) (Figure 4).

**Figure 5:**
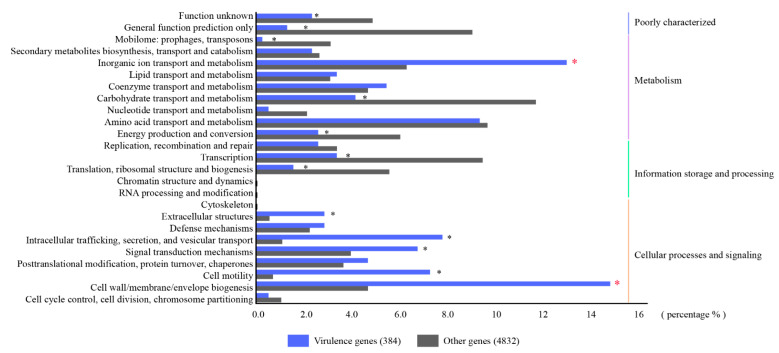
COG functional classification. COG functional annotation in virulence genes (blue rectangles) and other genes (gray rectangles). * represents *p* value was less than 0.01.

To reveal the genes related to the hypervirulence characteristics of strain HvKp-su1, virulence factors were identified according to the VFDB database. Strain HvKp-su1 had 396 virulence factors, with 391 virulence factors located on the chromosome and 5 virulence factors located on plasmid 1. We conducted COG functional classification analysis to reveal the functions of virulence genes. According to the results, the numbers related to “cell wall/membrane/envelope biogenesis” and “inorganic ion transport and metabolism” in virulence genes were significantly higher than those in other genes, with 14.8% vs. 4.70% and 13.0% vs. 6.31%, respectively (Figure 5). These findings indicated that genes involved in the function of “cell wall/membrane/envelope biogenesis” and “inorganic ion transport and metabolism” are the key determinants of virulence of strain HvKp-su1.

**Figure 6:**
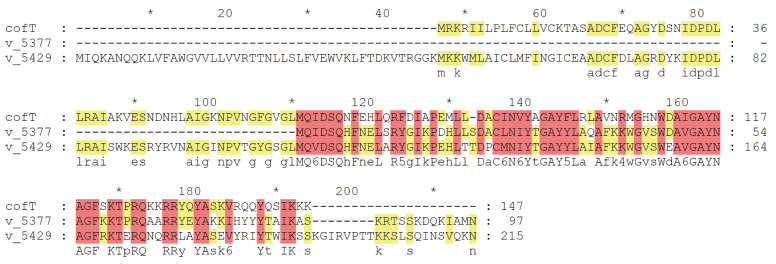
Amino acid sequence alignment of v_5377 and v_5429 to *cofT.* Homologous analysis of v_5377 and v_5429 in strain HvKp-su1 and *cofT* in *E. coli.*

Based on well-known studies, multiple virulence genes on chromosomes are considered hypervirulent, including genes belonging to “inorganic ion transport and metabolism”: aerobactin (*iutA*), salmochelin (*iroEN*), yersiniabactin (*ybtAEPQSTUX*), and the type 3 fimbriae-encoding system (*mrkABCDF*). In addition, five virulence genes, v_5377 (*cofT*), v_5429 (*cofT*), v_5458 (*hopAN1*), v_5459 (*hopAN1*), and v_5486 (*virB*), were predicted on plasmid 1 (Figure 5) (Table 2). Among these five genes, v_5377 and v_5429 belong to the COG function of “cell wall/membrane/envelope biogenesis” and share relatively high identities (58.54% and 53.19%) with colonization factor antigen III, CFA/III lytic transglycosylase cofT in *Escherichia coli* (GenBank number: AB049751.1), which is also known as a major virulence determinant for the pathogenesis of *K. pneumoniae* (Figure 6). To further confirm whether strain HvKp-su1 carries the *rmpA*, *rmpA* 2, and *magA* genes, we performed a polymerase chain reaction (PCR) for the *rmpA*, *rmpA* 2, and *magA* genes as described previously. The results showed that *rmpA*, *rmpA* 2, and *magA* were not detected in this isolate.

Additionally, forty-eight genes of strain HvKp-su1 were predicted to be antimicrobial resistance genes, including genes of resistance to fosfomycin (*fosA*), kasugamycin (*KsgA*), sulfonamide (*sul1*), vancomycin (*vanA*), tetracycline (*tet*), trimethoprim (*dfrA22*), β-lactams (*bla*_TEM-1_, *bla*_SHV-55_, *bla*_CTX-M-65_), and the carpbapenemase gene *bla*_KPC-2_. The *bla*_TEM-1_, *bla*_SHV-55_, *bla*_CTX-M-65_, and *bla*_KPC-2_ genes were located on plasmid 1, and the other genes were located on the chromosome (Table 2).

## 3. Discussion

In this study, we described the clinical and molecular characteristics of an ST11-K47 CR-hvKP strain, HvKp-su1, in a patient who presented with a liver abscess combined with diabetes, pneumonia, pleural infection, abdominal abscess, and splenic abscess.

Our results demonstrated that although HvKp-su1 showed a negative “string” test, lacking the *rmpA*, *magA*, and pLVPK plasmids, it showed high virulence for the following reasons. First, the patient infected with HvKp-su1 developed multisite infections, including pneumonia, pleural effusion, liver abscess, and splenic abscess. *K. pneumoniae* cultured from five sites (shunt fluid of abdomen, shunt fluid of spleen fossa, abdominal fluid, catheter, and pleural fluid) had the same sequence type with strain HvKp-su1 (drainage fluid of the liver abscess). Second, strain HvKp-su1 caused a high mortality rate (90%) in *G. mellonella* larvae, as high as the hypervirulent strain ATCC43816 (91.67%). Third, the sequences of chromosome and plasmid 1 in strain HvKp-su1 are very similar to *K. pneumoniae* strain JX-CR-hvKP-2, which is a CR-hvKP strain owning a type of pLVPK-like plasmid and having a 50% lethal dose (LD50) < 10^5^ CFU to mice [15].

The positive “string test” is usually used to identify hvKP, but recent studies have demonstrated its poor performance in distinguishing hvKP [16]. Virulence factors, such as the mucoviscosity-associated gene A *magA*, capsular polysaccharide synthesis regulator gene *rmpA*, and iron uptake gene *kfu,* have been believed to also be significantly associated with the hypermucoviscosity and high virulence of hvKP [17,18,19]. However, HvKp-su1 showed a negative “string test” and lacked the virulent genes mentioned above. According to a new study, some CR-hvKP strains have lost the *rmpA* and *rmpA2* genes owing to the increased fitness cost of coding essential virulence factors and antimicrobial resistance [20]. At the same time, compared with *rmpA*, aerobactin (*iutA*) has been proven to be a stable marker for defining hvKP [9,20]. Aerobactin (*iutA*), together with salmochelin (*iroEN*) and yersiniabactin (*ybtAEPQSTUX*) in HvKp-su1are siderophore genes, are utilized by the strain to acquire Fe from the host to survive during infection [21]. These genes may play important roles in the virulence process of HvKp-su1. In addition, according to the VFDB database, five genes located on the plasmid, v_5377, v_5429, v_5458, v_5459, and v_5486, were also predicted to be virulence genes in HvKp-su1. Compared with the other three uncharacterized genes, v_5377 and v_5429 belong to the COG class of “cell wall/membrane/envelope biogenesis” and have a high identity with VFG042772 (*cofT*, colonization factor antigen III, CFA/III (CS8)) (VFDB analysis), *Salmonella enterica* serovar Typhimurium LT2 (COG analysis), lysozyme-related protein Hpa2 (KEGG analysis), which was also reported as the main reason for the high virulence of Hv-CRKp strain R16 [22]. CofT has a lysozyme-like domain and produces type 4b pilus CFA/III lytic transglycosylase. CFA/III is a colonization factor antigen (CFA) group that assists the release of enterotoxins by attaching to the epithelium of the small intestine in *Enterotoxigenic Escherichia coli* (ETEC) [23,24]. Its homology genes, IagB of *Salmonella enterica* serovar Typhi and IpgF of *S. flexneri*, participate in the invasion of eukaryotic host cells [25].

Several genes encoded for resistance are associated with ST11, such as NDM and KPC [26,27]. In China, ST11 with a KPC-2-producing clone is a common type of CR-hvKP [28]. K1, K2, and K57 were the most common serotypes of CR-hvKP. However, the ST11-K47 CR-hvKP in this study has rarely been reported. In a report in 2020, Qi Wen Yang et al. demonstrated that ST11-K47/K64 Hv-CRKp was the main serotype causing bacterial liver abscesses in China, which highlights the importance of controlling ST11-K47 CR-hvKP [22].

*K. pneumoniae* may erode to the liver through contiguous spread from the infection of adjacent tissue. There is evidence that *K. pneumoniae* colonizes the intestine before causing a liver abscess [29,30]. Overuse of antibiotics, such as ampicillin, which disrupt intestinal microbiota but have little effect on *K. pneumoniae*, may promote its invasion into the liver [31]. In this study, moxifloxacin and meropenem were used before *K. pneumoniae* was detected, which may induce an imbalance of intestinal flora and promote the invasion of *K. pneumoniae*.

In addition to the expression of the virulence factors of microbes, host risk factors such as diabetes are believed to be significant risk factors for *K. pneumoniae* pyogenic liver abscesses (KPLA). A total of 43% of KPLA patients are diagnosed with diabetes, which indicates that diabetes may promote the development of liver abscesses [32]. Compared with patients with controlled glycemia, patients with poor glycemic control tend to have more cryptogenic liver abscesses and metastatic infections [33]. A possible reason is that poor glycemic control may disrupt neutrophil phagocytosis of *K. pneumoniae* and impair the host’s defense mechanisms [34].

KPC-2 and CTX-M-65 were found to be the most dominant extended-spectrum β-lactamase (ESBL) genes in *K. pneumoniae* in China, especially in strain KL47 [35]. Available options to treat this CRKP are limited to tigecycline and polymyxin B. Since the nephrotoxicity of polymyxin is high, tigecycline was used in this case. According to reviews, tigecycline has good performance in community-acquired pneumonia (CAP) and complicated intra-abdominal infections (cIAIs) caused by CRE [36,37]. The combination regimen of tigecycline and meropenem used in this case is warranted when infections are caused by CRE and other carbapenem-sensitive organisms. Except for systemic antibiotic usage, percutaneous and surgical drainage are extremely necessary for patients who suffer from liver abscesses. Percutaneous drainage should be considered first due to its low risk and low cost, and surgical drainage is more suitable for patients with multiple or large abscesses [38].

## 4. Materials and Methods

### 4.1. Bacterial Identification and Antimicrobial Susceptibility Testing

Bacterial identification was carried out using a VITEK 2 automated instrument (bioMérieux, Marcy l’Etoile, France). Antimicrobial susceptibility was determined by the microdilution method. Minimum inhibitory concentrations (MICs) were interpreted according to interpretive standards of the Clinical and Laboratory Standards Institute (CLSI).

### 4.2. “String Test” Phenotypic Detection

*K. pneumoniae* was cultured on blood agar at 37 °C. The “string test” was negative if the length of the viscous string was <5 mm when pulled away from the colony with an inoculation loop.

### 4.3. G. mellonella Larvae Infection Assay

Pathogen-free, 300–350 mg, body length of 25–30 mm *G. mellonella* larvae were obtained from Huiyude Biotech Company, Tianjin, China. Twenty larvae were used as a sample population. Bacteria were grown in LB and harvested in the exponential phase. After being washed with PBS, 4 μL of bacterial suspension (with a concentration of 2 × 10^6^ CFU/mL) was injected into the last right proleg by a microsample syringe. PBS and ATCC43816 were used as the negative and positive control groups, respectively. Larvae were placed in sterile dishes with food and kept at 37 °C. Mortality rates were recorded for 60 h [39,40]. All experiments were repeated in biological triplicate. GraphPad Prism 7.0 was used for analysis and graph construction.

### 4.4. Whole-Genome Sequencing and Bioinformatics Analysis

The *K. pneumoniae* genome HvKp-su1 was sequenced using a PacBio RS II platform and Illumina HiSeq 4000 platform at the Beijing Genomics Institute (BGI, Shenzhen, China). PacBio subreads (length < 1 kb) were removed. The program Pbdagcon (https://github.com/PacificBiosciences/pbdagcon, last accessed 9 July 2020) was used for self-correction. Draft genomic unitigs, which are uncontested groups of fragments, were assembled using the Celera Assembler against a high-quality corrected circular consensus sequence subread set. General function annotation was conducted with the COG (Clusters of Orthologous Groups) database. The accession number of HvKp-su1 is PRJNA824690.

COG (Clusters of Orthologous Groups) and KEGG (Kyoto Encyclopedia of Genes and Genomes) were used for general function annotation. Virulence factors and resistance genes were identified based on the core dataset in the VFDB (Virulence Factors of Pathogenic Bacteria) and ARDB (Antibiotic Resistance Genes Database) databases. SPSS 22.0 was used for statistical analysis. A chi-square test or Fisher’s exact test was conducted to examine the functional distribution of virulent genes and other genes. Statistical significance was determined if a two-tailed p value was less than 0.01.

The alignment of the HvKp-su1 chromosome and plasmid 1 sequence with other hvKP genomes was performed using BLAST Ring Image Generator (BRIG).

Homologous amino acid sequence analysis of v_5377, v_5429, and *cofT* in *Escherichia coli* (GenBank number: AB049751.1) was performed with MEGA7. GeneDoc was used for graph construction.

### 4.5. Sequence Types (STs) and Capsular Type Analysis

STs and capsular types of HvKp-su1 and other *K. pneumoniae* (HvKp-su2, HvKp-su3, HvKp-ab, HvKp-ca, and HvKp-pf) were determined based on Multilocus sequence typing (MLST) (http://bigsdb.pasteur.fr/klebsiella/ last accessed 9 July 2020). Briefly, gapA, infB, mdh, pgi, phoE, rpoB, tonB, and wzi genes were amplified by PCR and sequenced. The sequence types (STs) and capsular types were identified by the MLST database [39]. Su2: shunt fluid of abdomen, su3: shunt fluid of spleen fossa, ab: abdominal fluid, ca: catheter, pf: pleural fluid.

## 5. Conclusions

In this study, we characterized the clinical and genetic basis of a rare ST11-K47 CR-hvKP strain (HvKp-su1) with multidrug resistance and hypervirulence phenotypes isolated from a patient with a serious liver abscess, which will offer valuable insights into the prevention and treatment of this infection. HvKp-su1 possesses a pLVPK-like plasmid (HvKp-su1 plasmid1) with *bla*_KPC-2_, *bla*_TEM-1_, *bla*_SHV-55_, *bla*_CTX-M-65_ genes and the predicted virulence gene *cofT*, making it an antimicrobial and hypervirulent threat to public health. The traditional definition of hvKP is not accurate, and more hypervirulence-associated factors require further study in the identification of hvKP. More work is needed to evaluate the function of new hypervirulence factors such as *cofT* in the process of the pathogenesis of ST11-K47 CR-hvKP.

## Figures and Tables

**Table 1:** Clinical presentation and laboratory findings.

Characteristics	Results
Age	67
Sex	Male
Fasting glucose, mmol/L	12.8
CRP, mg/L	76.5
Serum albumin, g/L	29
Platelets, ×10^9^/L	49
WBCs, ×10^9^/L	13
RBC, ×10^12^/L	2.9
Hemoglobin, g/L AST, U/L ALT, U/L GGT, U/L TBIL, μmol/L DBIL, μmol/L ALB, g/L CHE, U/L	85.00 30 16 114 29.2 20.8 29 1129
Rivalta test (ascites)	Positive
Hospital stay time	56 days
Fever time	About two months

**Table 2:** Genome information of the ST11-K47 CR-hvKP strain HvKp-su1.

Name	Genome Size (bp)	GC Content	Coding Genes	Virulence Genes	Drug Resistance Genes
HvKp-su1 Chromosome	5,514,909	57.55%	5358	*iutA*, *iroEN*, *ybtAEPQSTUX*, *mrkABCDF*	*fosA*, *KsgA*, *sul1*, *vanA*, *tet*, *dfrA22*
HvKp-su1 plasmid1	133,346	53.23%	143	v_5377, v_5429, v_5458, v_5459, v_5486	*bla*_TEM-1_, *bla*_SHV-55_, *bla*_CTX-M-65_, *bla*_KPC-2_
HvKp-su1 plasmid2	10,061	55.05%	12	/	/

## Data Availability

Publicly available datasets were analyzed in this study. This data can be found here: [SUB11310797-Summary|WGS|Submission Portal (nih.gov)/accession number: PRJNA824690.

## Abbreviations

Classic *Klebsiella pneumoniae*	(cKP)
Carbapenem-resistant classic *Klebsiella pneumoniae*	(CR-cKP)
Hypervirulent *Klebsiella pneumoniae*	(hvKP)
Carbapenem-resistant *Klebsiella pneumoniae*	(CR-KP)
Carbapenem-resistant hypervirulent *Klebsiella pneumoniae*	(CR-hvKP)
